# An Audit of Baseline Physical Health Monitoring in Patients Under the Care of Mersey Care NHS Foundation Trust Who Are Commenced on Lithium

**DOI:** 10.1192/bjo.2024.577

**Published:** 2024-08-01

**Authors:** Declan Hyland, Gopal Chinnari, Tawfik Elhaj-Houssen, Rose-Anne Orrell

**Affiliations:** Mersey Care NHS Foundation Trust, Liverpool, United Kingdom

## Abstract

**Aims:**

Lithium is clinically indicated for use in the UK for treatment and prophylaxis of mania, treatment and prophylaxis of bipolar disorder, treatment and prophylaxis of recurrent depressive disorder and treatment and prophylaxis of aggressive or self-harming behaviour. Prior to commencing lithium, there is a need for several physical health checks and blood tests to be completed to ensure that lithium remains appropriate to prescribe.

This audit aimed to establish whether Mersey Care NHS Foundation Trust’s prescribing practices of lithium are in keeping with national guidance prior to initiation and how the Trust’s performance compared with national performance as identified by the Prescribing Observatory for Mental Health (POMH) lithium audit.

**Methods:**

A total of 127 patients under the care of the Trust who were prescribed lithium (lithium carbonate and lithium citrate, tablet and liquid formulations) were identified using the Trust's electronic record system and electronic prescription chart system. The POMH lithium audit tool was used to capture data for each lithium patient as Mersey Care NHS Foundation Trust was participating in the national POMH lithium audit. Each patient's electronic record was scrutinised to determine whether the following were measured prior to lithium being initiated – weight/body mass index (BMI)/waist circumference, Thyroid Function Tests (TFTs), serum calcium level and estimated Glomerular Filtration Rate (eGFR).

**Results:**

Of the sample of lithium patients included in the audit, 78% of patients had a weight/BMI/waist circumference done prior to initiation of lithium; 80% of patients had a serum calcium level; 93% had TFTs done; and 100% of patients had an eGFR completed prior to initiation of lithium.

**Conclusion:**

The results of this audit indicate that the Trust is performing well with the required physical health monitoring prior to initiation of lithium. Trust performance for all four parameters that were included and assessed in this audit were above the national compliance level reported in the POMH lithium audit. There is clearly a need, however, to improve performance and to ensure that both medical and nursing staff across the Trust are aware of the physical health monitoring required before initiating any patient on lithium. A Quality Performance Alert will be sent to all medical and nursing staff to raise awareness and lithium monitoring will be included in the induction for junior doctors working in the Trust. Future auditing of Trust performance on required physical health monitoring prior to commencing lithium will be conducted.

